# An observational cohort study on shortened dental arches—clinical course during a period of 27–35 years

**DOI:** 10.1007/s00784-012-0765-6

**Published:** 2012-06-29

**Authors:** Anneloes E. Gerritsen, Dick J. Witter, Ewald M. Bronkhorst, Nico H. J. Creugers

**Affiliations:** 1 Department of Oral Function and Prosthetic Dentistry, College of Dental Science, Radboud University Nijmegen Medical Centre, Philips van Leydenlaan 25, 6525 EX Nijmegen, The Netherlands; 2 Department of Preventive and Restorative Dentistry, College of Dental Science, Radboud University Nijmegen Medical Centre, Philips van Leydenlaan 25, 6525 EX Nijmegen, The Netherlands

**Keywords:** Shortened dental arches, Complete dental arches, Cohort study, Removable denture prosthesis, Tooth loss, Clinical course

## Abstract

**Objectives:**

The objective of this study was to investigate the clinical course of shortened dental arches (‘SDA group’) compared to SDAs plus removable denture prosthesis (‘SDA plus RDP group’) and complete dental arches (‘CDA group’, controls).

**Materials and methods:**

Data (numbers of direct and indirect restorations, endodontic treatments, tooth loss and tooth replacements) were extracted from patient records of subjects attending the Nijmegen Dental School who previously participated in a cohort study on shortened dental arches with three to four posterior occluding pairs (POPs).

**Results:**

Records of 35 % of the original cohort were retrievable. At the end of the follow-up (27.4 ± 7.1 years), 20 out of 23 SDA subjects still had SDA with 3–4 POPs compared to 6 out of 13 for SDA plus RDP subjects (follow-up 32.6 ± 7.3 years). Sixteen out of 23 CDA subjects still had CDA; none of them lost more than one POP (follow-up 35.0 ± 5.6 years). SDA group lost 67 teeth: 16 were not replaced, 16 were replaced by FDP and 35 teeth (lost in three subjects) replaced by RDP. Mean number of treatments per year in SDA subjects differed not significantly compared to CDA subjects except for indirect restorations in the upper jaw.

**Conclusion:**

Shortened dental arches can last for 27 years and over. Clinical course in SDA plus RDP is unfavourable, especially when RDP-related interventions are taken into account.

**Clinical relevance:**

The shortened dental arch concept seems to be a relevant approach from a cost-effective point of view. Replacement of absent posterior teeth by free-end RDP cannot be recommended.

## Background

The shortened dental arch concept is a potentially cost-effective approach in the management of reduced dentitions. This concept is globally accepted, but not widely practiced [1]. A body of mainly circumstantial evidence shows that shortened dental arches, comprising all anterior teeth and three to five occluding units, provide a stable and functional dentition with respect to chewing ability, aesthetics and oral comfort [2–5]. The functionality of shortened dental arches has been reflected in outcomes of studies on oral health-related quality of life (OHRQoL). The outcomes are rather controversial: on the one hand, shortened dental arches are found to be related to OHRQoL impairment [6], especially when first molar contacts were absent [7] and on the other hand, subjects with a shortened dental arch reported to be satisfied with their oral status [8].

Oral health care aims at the retention of at least a functional and natural dentition throughout life. Therefore, besides functionality, longevity of shortened dental arches should also be taken into account when considering application of the shortened dental arch concept. The fact that shortened dental arch subjects have lost molars in the past implicitly indicates an increased risk for dental diseases in these subjects. It is reasonable to expect that this predisposition to dental diseases is not eliminated after applying the shortened dental arch concept and hence necessitate more treatment in course of time including additional tooth extractions. Further tooth loss may endanger the longevity of the shortened dental arch status and thus compromise oral function.

The consequences of this predisposition might be even more manifest in subjects with a shortened dental arch restored with removable dental prosthesis (RDP) because RDPs have been associated with increased incidence of caries and periodontal breakdown [9, 10]. However, a randomised clinical trial revealed no statistically significant difference in frequency of tooth loss after 3 years of follow-up among shortened dental arch subjects with and without RDP [11]. Furthermore, quality of life levels of subjects with a shortened dental arch with RDP were found to be almost identical to those of subjects with a shortened dental arch without RDP [12]. In another study, subjects with a shortened dental arch only perceived benefits of RDP from a OHRQoL perspective if anterior teeth replacements are included [6].

To our knowledge, the longest follow-up reports on shortened dental arches are based on a 9-year observational cohort study (3-, 6- and 9-year observations) [13–15]. It was concluded that shortened dental arches could provide a functional dentition with long-term occlusal stability [13, 14]. Furthermore, the study reported similar frequencies of signs and symptoms of temporomandibular disorders for shortened dental arches with and without RDP and complete dental arches (CDA) [15]. The present study is evaluating the initial cohort by analysing 27 to 35 years follow-up data on the basis of information from patient records. The objective of this study was to evaluate the clinical course of shortened dental arches by (1) assessing its longevity; (2) investigating the management of tooth loss and (3) analysing interventions provided during the follow-up period.

It was hypothesised that shortened dental arches have shorter longevity and receive more restorative interventions and tooth extractions than CDA. Moreover, it was hypothesised that these effects are more prone in shortened dental arches plus RDP.

## Materials and methods

### Data collection 

For this study, data from patient records of subjects who participated in a prospective observational cohort study on shortened dental arches (SDA) were used. The subjects from this cohort were regular patients of the Nijmegen Dental School Clinic for checkups and all necessary dental treatments. The initial cohort, a convenient sample, comprised subjects with shortened dental arches in at least one jaw with three to four posterior occluding pairs (POPs) and intact anterior areas without distal extension RDP (‘SDA group’, *n* = 74), subjects with a shortened dental arch extended by RDP (‘SDA plus RDP group’, *n* = 25), and subjects with CDA (‘CDA group’, *n* = 72, control group). Teeth replaced by fixed dental prostheses (FDPs) were considered as present; occluding posterior FDP replacements were counted as POPs. Detailed information on the sampling method can be found in a previously published report [13].

For the present study, data were extracted from the available patient records of the subjects of the initial cohort. All restorative interventions provided from the time the subjects had subscribed at the dental school until May 2011, or as long as information was available, were extracted from these records. From the moment dentitions were identified as SDA or SDA plus RDP, the following interventions were considered: direct restorations (fillings), indirect restorations (crowns, in- and onlays), endodontic treatments, and tooth extractions.

To be able to describe the changes in functional status of the dentitions, the total number of lost teeth (excluding third molars) and the number of POPs at baseline and endpoint were determined (Table 2). Also, tooth replacements were recorded, including type of replacements (resin bonded FDP, conventional FDP, partial RDP and complete dentures) and location in the dental arch.

If subjects became edentulous or if dental records were closed (i.e. subjects decided to stop attending the dental school or died), data recording ceased. Accuracy of the patient records was checked by information of available X-rays and data from the 9-year follow-up observations of the original cohort study.

### Statistical analysis

Mean age at baseline of CDA subjects was significantly higher than that of SDA subjects (*p* value of 0.045). Because comparing groups with a *t* test cannot eliminate ‘age’ as a potential confounding variable, the groups were compared using linear regression analyses with age and ‘group’ as independent variables. Since the variable age is only used to eliminate confounding from all regression analyses, only the effect (i.e. the difference corrected for age) of the variable group is reported. ‘Gender’ was also checked for being a potential confounding variable. The group effects with or without gender in the multivariate model were nearly identical, so gender is not a confounder in this study. It appeared that in less than 10 % of the analyses, gender had an effect and these effects were extremely small compared to the group effects. Therefore, it was decided to not include gender in the models presented. ‘SDA’ was compared with SDA plus RDP and with CDA. For the analyses presented in Tables 3 and 4, third molars were taken into account to be able to get insight in the total number of treatments provided in the groups. First, baseline and endpoint status regarding numbers of teeth with direct and indirect restorations and absent teeth were compared. Next, the number of direct and indirect restorations made and the endodontic treatments and tooth extractions provided per year were analysed for each group.

Finally, to get insight in which dental regions most interventions were needed, the number of direct restoration provided per tooth per year for the ‘anterior region’, ‘premolar region’ and ‘molar region’ was analysed. For these analyses, in which the third molars were excluded, only the time teeth were actually present was taken into account and for the molar region only SDA subjects having molars at baseline were analysed. The statistical analyses were performed using SPSS 18.0 software.

## Results 

### Sample

Eventually, patient records of 59 subjects of the original cohort (35 %) appeared to be retrievable from the database of the dental school. Table 1 presents mean age at baseline, gender distribution and mean time of follow-up of each group (SDA, SDA plus RDP and CDA).

**Table 1:** Number of subjects, mean age at baseline (SD), gender distribution and mean time of follow-up (SD) of the SDA group, SDA plus RDP group and CDA group (control)

	SDA	SDA plus RDP	CDA
N (% of original cohort)	23 (31.1)	13 (52.0)	23 (31.9)
Mean age at baseline (SD)	37.8 (11.2)	40.0 (9.7)	31.7 (8.0)
Male %	21.7	38.5	47.8
Mean time of follow-up	27.4 (7.1)	32.6 (7.3)	35.0 (5.6)

### Clinical course

The majority (87 %) of the subjects in the SDA group still had an SDA status comprising at least three POPs at the end of the follow-up period (mean 27.4 years, SD 7.1), despite the loss of 67 teeth in this group during this period (Table 2). Three subjects, accountable for 52 % of the 67 lost teeth, lost their SDA status: one subject became edentulous and in two subjects shortened dental arches became interrupted dental arches (IDA). In the remaining 20 SDA subjects, FDPs replaced 16 lost teeth, thereby maintaining the SDA status of the subjects. Another 16 lost teeth were not replaced, of which 12 teeth (75 %) had no opposing tooth.

**Table 2:** Clinical course of SDA, SDA plus RDP and CDA subjects; tooth loss (excluding third molars) and management of tooth loss during follow-up period and dental functional status at end of follow-up

Dental status at baseline	SDA (*n* = 23)	SDA plus RDP (*n* = 13)	CDA (*n* = 23)
Tooth loss and management of tooth loss	Tooth loss:	Tooth loss:	Tooth loss:
	67 teeth lost in 17 subjects (6 subjects had no tooth loss)	63 teeth lost in 12 subjects (1 subject had no tooth loss)	15 teeth lost in 10 subjects (13 subjects had no tooth loss)
	Management:	Management:	Management:
	• 16 teeth not replaced (24 %)	• 11 teeth not replaced (17 %)	• 7 teeth not replaced (47 %)
	• 16 teeth by fixed replacement (24 %):	• 3 teeth by fixed replacement (5 %):	• 8 teeth by fixed replacement (53 %):
	Conventional FDP, 11	Conventional FDP, 3	Conventional FDP, 6
	Resin-bonded FDP, 1		Resin-bonded FDP, 1
	Implant, 4		Implant, 1
	• 35 teeth replaced by RDP (52 %):	• 49 teeth replaced by RDP (78 %):	• No RDP replacement (0 %)
	Acrylic RDP, 8 teeth (in 2 RDPs)	Metal frame RDP, 27 (in 8 RDPs)	
	Complete denture, 27 teeth (in 1 CD)	Complete denture, 22 (in 1 CD)	
Dental functional status at end of follow-up and change in number of POPs	Dental status in subjects at end of follow-up:	Dental status in subjects at end of follow-up:	Dental status in subjects at end of follow-up:
	SDA, 20	SDA with RDP, 6	CDA, 16
	IDA with RDP, 2	SDA without RDP, 3	IDA, 3
	Edentulous, 1	IDA with RDP, 3	SDA (slightly), 4
		Edentulous, 1	
	POPs change in subjects:	POPs change in subjects:	POPs change in subjects:
	• Lost POPs:	• Lost POPs:	• Lost POPs:
	None, 16	None, 3	None, 16
	1–2, 4	1–2, 4	1, 7
	All, 1	3, 1	

		All, 3	
	• Gained POPs:	• Gained POPs:	• Gained POPs:
	1, 2	1–2, 2	n.a.

*n.a.* not applicable

At the end of the follow-up period, the functional status of the subjects of the SDA plus RDP group is far more diverse than for subjects of the SDA group. After a mean follow-up time of 32.6 years (SD 7.3), fewer than half of the subjects (46 %) still had the status SDA plus RDP. Only three subjects (23 %) retained all their POPs. Proportionally, more teeth were lost in this group than in the SDA group (63 teeth in 13 SDA plus RDP subjects vs. 67 teeth in 23 SDA subjects). Generally, the replacement of lost teeth in the SDA plus RDP group was accomplished by (adding teeth to a present) RDP (78 % of the lost teeth). Four out of seven subjects who lost their SDA plus RDP status did so because of further tooth loss: one subject became edentulous and three lost their status because of dental arch interruptions and loss of POPs. The other three subjects lost their SDA plus RDP status because two of them had their RDPs replaced by free-end FDP and one subject ceased wearing the RDP (Table 2).

Seventy percent of CDA subjects maintained their status at the end of the follow-up period (35.0 years, SD 5.6). The number of teeth lost was relatively low in this group compared to the SDA group (15 teeth in 23 CDA subjects vs. 67 teeth in 23 SDA subjects). None of the subjects lost more than one POP (Table 2).

### Interventions

#### Comparison of SDA with SDA plus RDP

At baseline, there were no statistically significant differences in the mean number of teeth with direct restorations and indirect restorations between the SDA group and the SDA plus RDP group (Table 3). Considering absent teeth at baseline, the SDA group had more absent teeth in the upper jaw but fewer absent teeth in the lower jaw compared to the SDA plus RDP group. At the end of follow-up, both SDA group and SDA plus RDP group had fewer teeth with direct restorations in the upper jaw than at baseline. This decrease is explained by the increase of the number of teeth with indirect restorations (direct restorations were replaced by indirect restorations) and the increase of the number of absent teeth.

**Table 3:** Number of teeth with direct restorations and indirect restorations and number of absent teeth for upper and lower jaw for SDA, SDA plus RDP and CDA subjects at baseline and end of follow-up (mean number (SD))

Status per jaw		SDA (*n* = 23)	SDA plus RDP (*n* = 13)	CDA (*n* = 23)	Comparison between SDA and SDA plus RDP			Comparison between SDA and CDA		
					Effect	*p* value	95 % CI	Effect	*p* value	95 % CI
Direct restorations										
Upper	Baseline	5.74 (3.74)	6.00 (3.96)	6.43 (3.32)	−0.48	0.716	(−3.14, 2.78)	−0.58	0.602	(−2.81, 1.65)
	Endpoint	4.00 (3.22)	3.23 (2.09)	6.87 (3.75)	0.76	0.469	(−1.31, 2.83)	−2.97	0.009	(−5.16, −0.77)
Lower	Baseline	3.39 (2.39)	2.92 (2.02)	5.83 (2.02)	0.41	0.602	(−1.99, 1.17)	−3.70	<0.001	(−5.07, −2.34)
	Endpoint	4.52 (2.13)	2.69 (1.80)	4.52 (2.13)	0.93	0.239	(−0.65, 2.50)	−1.32	0.064	(−2.72, 0.08)
Indirect restorations										
Upper	Baseline	0.35 (0.65)	0.08 (0.28)	0.22 (0.67)	0.28	0.160	(−0.12, 0.67)	0.07	0.719	(−0.34, 0.49)
	Endpoint	4.09 (3.01)	3.77 (3.19)	2.00 (2.47)	0.11	0.915	(−2.00, 2.22)	2.42	0.006	(0.72, 4.12)
Lower	Baseline	0.17 (0.58)	0.37 (0.86)	0.13 (0.34)	−0.10	0.682	(−0.58, 0.38)	0.03	0.840	(−0.32, 0.26)
	Endpoint	2.04 (2.48)	2.08 (1.44)	2.52 (2.17)	−0.16	0.833	(−1.67, 1.35)	0.19	0.791	(−1.63, 1.25)
Absent teeth										
Upper	Baseline	4.09 (1.88)	2.62 (2.10)	0.91 (0.95)	1.54	0.032	(0.14, 2.94)	2.89	<0.001	(2.00, 3.78)
	Endpoint	5.22 (2.78)	5.85 (4.10)	1.70 (0.82)	−0.44	0.697	(−2.75, 1.86)	3.28	<0.001	(2.01, 4.55)
Lower	Baseline	5.35 (1.15)	6.23 (0.83)	0.83 (0.94)	−0.92	0.017	(−1.67, −0.17)	4.54	<0.001	(3.88, 5.20)
	Endpoint	6.04 (2.25)	7.69 (3.23)	1.91 (0.73)	1.58	0.096	(−3.45, 0.30)	4.05	<0.001	(3.01, 5.09)

Effect, *p* value and 95 % CI are corrected for age

The mean numbers of indirect restorations, direct restorations, endodontic treatments and tooth extractions provided per year did not differ statistically among the SDA group and SDA plus RDP group (Table 4). Focusing on direct restorations provided in the different dental regions also revealed no statistical significant differences between the groups (Table 5). In the SDA group, the following RDP-related interventions were provided during the follow-up period: two partial RDPs and one complete denture; for the SDA plus RDP group, this was 26 replacement partial RDPs (all subjects received 1–4 new/replacement RDPs), 3 additional partial RDPs (in opposite jaw), 24 relinings, 13 repairs and 9 extensions to add lost teeth to a present RDP and one complete denture.

**Table 4:** Restorative interventions and tooth extractions provided per year in SDA, SDA plus RDP and CDA subjects for upper and lower jaw (mean number (SD))

Treatment per jaw	SDA (*n* = 23)	SDA plus RDP (*n* = 13)	CDA (*n* = 23)	Comparison between SDA and SDA plus RDP			Comparison between SDA and CDA		
				Effect	*p* value	95 % CI	Effect	*p* value	95 % CI
Direct restorations									
Upper	0.72 (0.36)	0.69 (0.41)	0.58 (0.42)	0.04	0.755	(−0.23, 0.32)	0.11	0.371	(0.14, 0.36)
Lower	0.38 (0.23)	0.42 (0.23)	0.42 (0.22)	-0.02	0.803	(−0.17, 0.13)	-0.07	0.308	(−0.21, 0.07)
Indirect restorations									
Upper	0.18 (0.16)	0.19 (0.14)	0.08 (0.09)	-0.02	0.744	(−0.13, 0.09)	0.11	0.008	(0.03, 0.19)
Lower	0.10 (0.13)	0.09 (0.06)	0.08 (0.06)	0.00	0.914	(−0.07, 0.08)	0.04	0.221	(−0.03, 0.10)
Endodontic treatments									
Upper	0.05 (0.05)	0.07 (0.08)	0.03 (0.04)	-0.02	0.407	(−0.06, 0.03)	0.02	0.154	(−0.01, 0.05)
Lower	0.03 (0.04)	0.03 (0.04)	0.02 (0.03)	-0.01	0.699	(−0.03, 0.02)	0.01	0.351	(−0.01, 0.03)
Tooth extractions									
Upper	0.06 (0.08)	0.12 (0.12)	0.03 (0.03)	-0.05	0.124	(−0.12, 0.02)	0.03	0.186	(−0.01, 0.06)
Lower	0.05 (0.10)	0.06 (0.10)	0.03 (0.03)	-0.05	0.878	(−0.08, 0.07)	0.84	0.610	(−0.04, 0.05)

Effect, *p* value and 95 % CI are corrected for age

**Table 5:** Direct restorations provided per year in SDA, SDA plus RDP and CDA subjects per tooth for upper and lower jaw (mean number (SD)) (third molars excluded)

Jaw	Region	SDA (*n* = 23)	SDA plus RDP (*n* = 13)	CDA (*n* = 23)	Comparison between SDA and SDA plus RDP			Comparison between SDA and CDA		
					Effect	*p* value	95 % CI	Effect	*p* value	95 % CI
Upper	Anterior	0.074 (0.049)	0.073 (0.049)	0.034 (0.046)	0.002	0.922	(−0.033, 0.037)	0.034	0.025	(0.005, 0.064)
	Premolar	0.062 (0.041)	0.038 (0.032)	0.042 (0.029)	0.024	0.077	(−0.003, 0.051)	0.019	0.098	(0.004, 0.041)
	Molar	0.025 (0.032)^a^	0.036 (0.034)^b^	0.053 (0.036)	−0.013	0.231	(−0.035, 0.009)	−0.025	0.021	(−0.047, 0.004)
Lower	Anterior	0.019 (0.026)	0.031 (0.026)	0.002 (0.003)	−0.010	0.247	(−0.027, 0.007)	0.012	0.022	(0.002, 0.022)
	Premolar	0.055 (0.035)	0.056 (0.037)	0.045 (0.038)	0.000	0.976	(−0.026, 0.025)	0.014	0.207	(−0.008, 0.037)
	Molar	0.011 (0.020)^c^	0.005 (0.009)^d^	0.056 (0.027)	0.008	0.147	(−0.003, 0.019)	−0.049	0.001	(−0.064, 0.034)

Effect, *p* value and 95 % CI are corrected for age. For the molar region, only SDA subjects having molars at baseline were included

^a^ *n* = 18

^b^ *n* = 13

^c^ *n* = 8

^d^ *n* = 5

#### Comparison of SDA with CDA (control)

Although the SDA subjects have, by definition, fewer teeth than the CDA subjects, the mean number of teeth with direct restorations and indirect restoration at baseline was not statistically significantly different between both groups, except for direct restorations in the lower jaw in the CDA group (Table 3). At the end of follow-up, the mean number of teeth with direct and indirect restorations was still not statistically significantly different for the lower jaw. For the upper jaw however, the number of direct restorations at the end of follow-up was lower in the SDA group compared to the CDA group (4.00 vs. 6.87) whereas the number of indirect restorations was higher in the SDA group (4.09 vs. 2.00, Table 3).

The mean numbers of indirect restorations, direct restorations, endodontic treatments and tooth extractions provided per year were only statistically significantly different for indirect restorations in the upper teeth (0.18 indirect restorations per year in SDA compared to 0.08 per year in CDA, *p* = 0.008) (Table 4).

For anterior teeth, statistically significant (upper jaw *p* = 0.025, lower jaw *p* = 0.022) more direct restorations per year were provided in the SDA group than in the CDA group (Table 5). There was no statistically significant difference in the number of direct restorations per year in premolars, whilst fewer direct restorations were made per year in molars in the SDA group compared to CDA group (upper jaw *p* = 0.021, lower jaw *p* = 0.001) (Table 5).

## Discussion 

This observational cohort study demonstrated that shortened dental arches could be preserved for periods of 27 years and over. On dentition level the number of restorative interventions provided per year was not significantly different among the groups, expect that ‘SDA’ received significantly more indirect restorations in the upper jaw than ‘CDA’. Partially this is due to the high number of abutment crowns needed for the high number of FDPs provided in SDA subjects. This indicates that on dentition level the cost of restorative treatment in a shortened dental arch is at least as high as the costs of restorative treatment in a CDA. However, on tooth level it was found that in SDA group compared to CDA group more direct restorations were provided per tooth per year, except for molars. A plausible explanation for these higher numbers of direct restorations per tooth per year in shortened dental arches is that a predisposition to dental diseases in this group, that caused the loss of molars in the first place, continues to have its effect on the remaining dentition. In course of time this will necessitate additional treatment since new carious lesions develop. However, the reasons for restorative treatment were often not available from the records.

Besides the initial restoration of carious lesions, fillings are also made to replace previous restorations for reasons such as fracture of the filling or secondary caries. A review of 10 surveys including 32.777 direct restorations revealed that more than 50 % of the provided restorations were replacements of previous restorations [16]. Therefore, it is assumable that considerable numbers of direct restorations provided in this cohort were replacement restorations. Hence, the high numbers of restorations provided per year per tooth in SDA subjects (Table 5) can probably partly be explained by the fact that SDA subjects already had relatively higher restoration levels (i.e. comparable numbers of teeth with restorations but in total significantly fewer teeth) at baseline than CDA subjects (Table 3). The only exception was the number of teeth with direct restorations in the lower jaw (5.83 in CDA group vs. 3.39 in SDA), which can be explained by the large difference in numbers of teeth present among the groups (0.83 teeth absent in CDA group vs. 5.35 in SDA).

Another explanation might be that in shortened dental arches fewer teeth have to bear the loads that occur during chewing. Consequently, increased loading of fewer teeth in shortened dental arches compared to CDAs possibly results in a higher failure rate of fillings in shortened dental arches. However, a study on masticatory performance showed that in shortened dental arches significantly lower occlusal forces could be measured than in complete dental arches [2]. In line with this, it is feasible that the higher number of direct restorations made in shortened dental arches is not due to overloading and subsequent failure of fillings.

For the present study, information from patient records from the dental school was used. If subjects incidentally visited a general dentist outside the dental school for treatment it is likely that relevant information is missing. In the Netherlands however, where this investigation was conducted, patients are generally loyal to their dentist and only seldom switch temporarily. Therefore we trust that the information in the dental school records is reasonably complete. However, to confirm the accuracy of the records, the data were compared with available X-rays and the investigation forms of the original cohort study.

The small sample size and selected group of dental school patients might limit external validity of this study, e.g. due to stricter maintenance protocols applied to these patients. On the other hand, we consider the quality of the data satisfactory for conclusive outcomes. Sixty-five percent of the dental records of the subjects of the original cohort were not retrievable, mainly of subjects who stopped attending the dental school. According to the Dutch legislation, patient files must be kept at least for 10 years from the moment patients unsubscribe. Fortunately, most files were kept longer, but still a reasonable number of archived files were destroyed. Probably most of these were destroyed at the time that the Nijmegen Dental School changed from paper to electronic patient files in 2003. The destruction of archived records seems to be rather arbitrary and as a result selection bias is considered absent or small.

The number of female subjects was proportionally high for the SDA group and SDA plus RDP group, whereas gender distribution for the CDA group was even (Table 1). This high percentage female subjects is a reflection of the dental school patient population. The CDA subjects, as being a control group, were selected by purpose sampling aiming at equal gender proportions. However analyses did not reveal gender effects and therefore correction for gender was not considered necessary.

Previous longitudinal studies on tooth loss reported mean numbers of 0.03 to 0.24 teeth lost per year [17, 18]. This is in accordance with incidence of tooth loss found in the present study; incidence varied from 0.03 to 0.12 teeth per year depending on location and group, with the lowest incidence for the lower jaw in CDA group and highest incidence for the upper jaw in SDA plus RDP group. In the majority of SDA subjects in the present study however, tooth loss did not lead to loss of their SDA status: a considerable number of lost teeth were molars without opposing tooth whilst lost teeth causing interruption of the dental arch were replaced by FDPs, by what means the SDA status was maintained. However, especially as incidence of tooth loss has been reported to increase with age, further tooth loss can be expected for this meanwhile aged cohort, which can endanger the functionality of the dentitions [18, 19].

It is striking that three of the 23 SDA subjects (these were also the subjects who lost their SDA status) were accountable for 52 % of the lost teeth. In contrast to this, 6 SDA subjects did not lose any tooth at all during the follow-up period. Apparently in these subjects the predisposition to dental diseases, as argued above, could be stopped or at least substantially decreased. To what extent and how risk factors for tooth loss exactly continue to have their effect in SDA subjects, is a phenomenon that needs further investigation.

At the start of the study it was hypothesised that adverse effects are more prone in SDA plus RDP subjects than in SDA subjects. However, it appeared that the number of tooth extractions per year was not statistically significant different. This is in accordance with the outcomes of a randomised clinical trial on tooth loss in shortened dental arches with or without RDP; Kaplan-Meier survival rates were not statistically significant different between the two groups in a 3-year follow up period [11]. Although the incidence of tooth loss is not significantly different between groups, 7 out of 13 SDA plus RDP subjects lost their SDA plus RDP status during the follow-up period whilst only 3 out of 23 SDA subjects lost their SDA status. This is also reflected in the loss of POPS; 62 % of the SDA plus RDP subjects lost one or more POP vs. 22 % of the SDA subjects. However, not all subjects lost their ‘SDA plus RDP’ status due to further tooth loss; three subjects lost this status because they stopped wearing their RDP and two of them actually gained POPs by having their RDPs replaced by free-end FDPs.

The number of restorative interventions for SDA plus RDP group provided per year during the follow period was not significantly different indicating that the costs at dentition level are the same as in SDA. When the costs related to RDP are taken into account, it can be concluded that the total costs in the SDA plus RDP group was higher whereas the longevity appeared to be lower compared to shortened dental arches without RDP. Additionally, apart from costs, every new RDP or RDP adjustment can bring discomfort and will make a considerable appeal on the adaptability of a patient. Moreover, it is questionable whether SDA patients actually benefit from RDP. As stated before, RDPs seem to contribute to OHRQoL only if anterior teeth replacements are included [6]. Furthermore, Aras et al. showed that RDPs in shortened dental arches did not improve masticatory performance [2] and McKenna et al. showed [20] that both prosthetic rehabilitation to a functional dentition as well as full rehabilitation including RDP did not improve the nutritional status as reflected in hematological markers. Several studies, including the present study, showed that RDPs often even have an adverse effect [10, 21–23]. In a randomised clinical trial on caries incidence following restoration of shortened lower dental arches in an elderly sample of patients, it was found that 2 years after restoration, there was a significantly greater incidence of new and recurrent caries lesions in subjects restored with RDPs compared with cantilever resin-bonded bridges [22]. In the same sample of elderly, it was found that subjects considered restoration with cantilever resin-bonded bridges more comfortable than restoration with RDP [23]. Also, a higher maintenance frequency for RDPs compared to resin-bonded FDPs in shortened lower dental arches was reported [10]. In summary, it can be stated that replacement of absent posterior teeth by a free-end RDP in a shortened dental arch is not recommendable; fixed appliances (cantilever (resin-bonded) FDP or implant supported FDP) might be preferable alternatives.

Recently, the body of evidence on the SDA concept was assessed by means of the Grading of Recommendation Assessment and Evaluation [24].The conclusion of this assessment was that the quality of the evidence for recommendation of the management of shortened dental arches is low because of the lack of evidence provided by randomised clinical trials [24]. However, conducting well-designed randomised clinical trials may be not feasible because of concerns of ethical and practical nature. Although the present study is not a randomised clinical trial, the strength of the present study is that it is a long-term clinical observational cohort study that provides valuable, long-term clinical data on the clinical course of shortened dental arches.

## Conclusions and clinical relevance

This study shows that shortened dental arches can last for 27 years and over. On dentition level, the number of treatments provided is comparable with complete dentitions. Herewith, this study contributes to the body of evidence that the SDA concept is a cost-effective approach. Moreover, replacement of absent posterior teeth by free-end RDP in shortened dental arches is not recommendable since RDP seems to be associated with a less favourable clinical course.
